# Supplementary figures and images for: Analyzing robust dividend payout policy with dynamic panel regression: Application of speed of adjustment to half-life
Source: PLoS One. 2025 Jan 15;20(1):e0316478. doi: 10.1371/journal.pone.0316478 (PMC11734913; doi:10.1371/journal.pone.0316478)

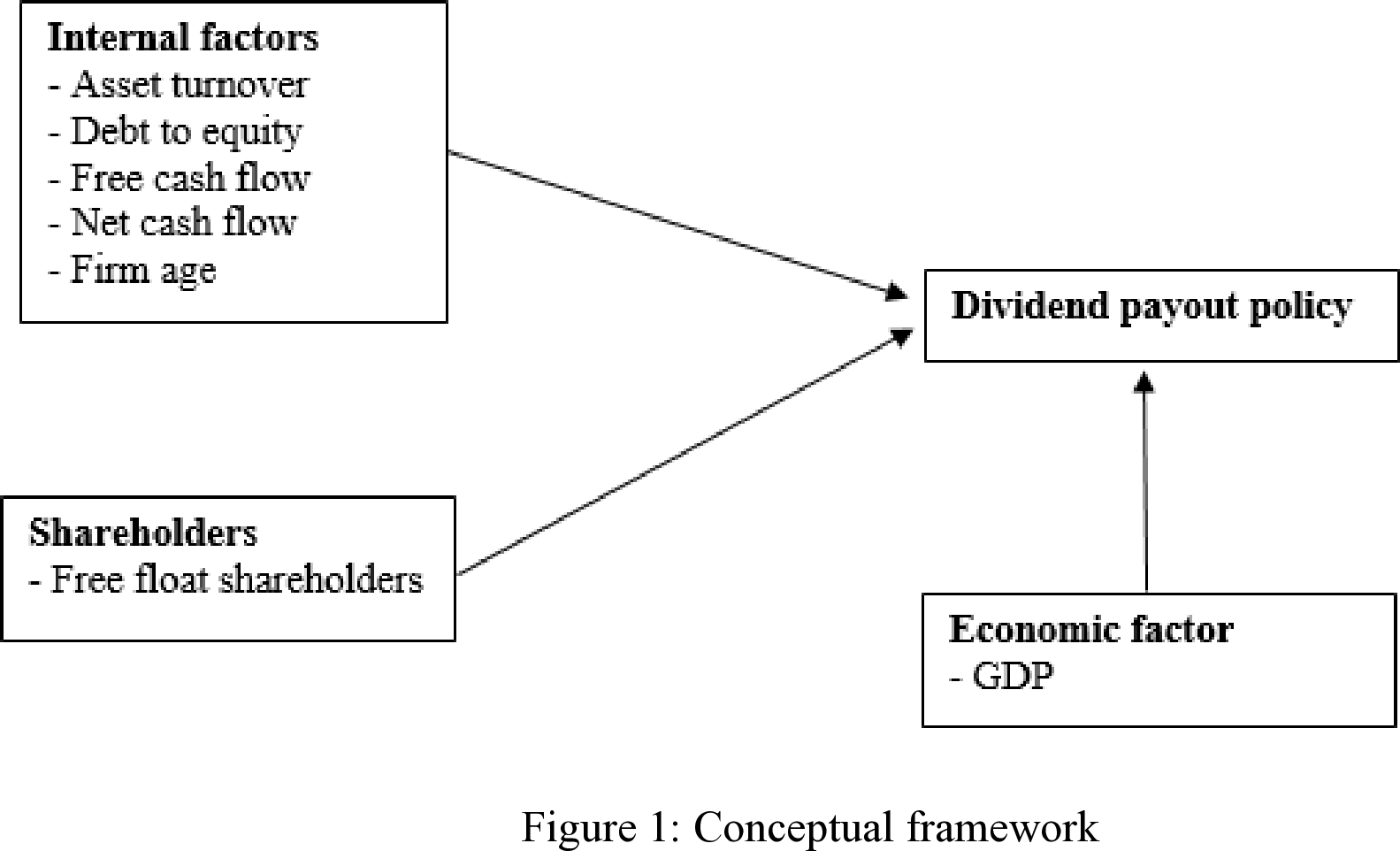

Supplement: S1 Fig — (TIF) [file pone.0316478.s001.tif]

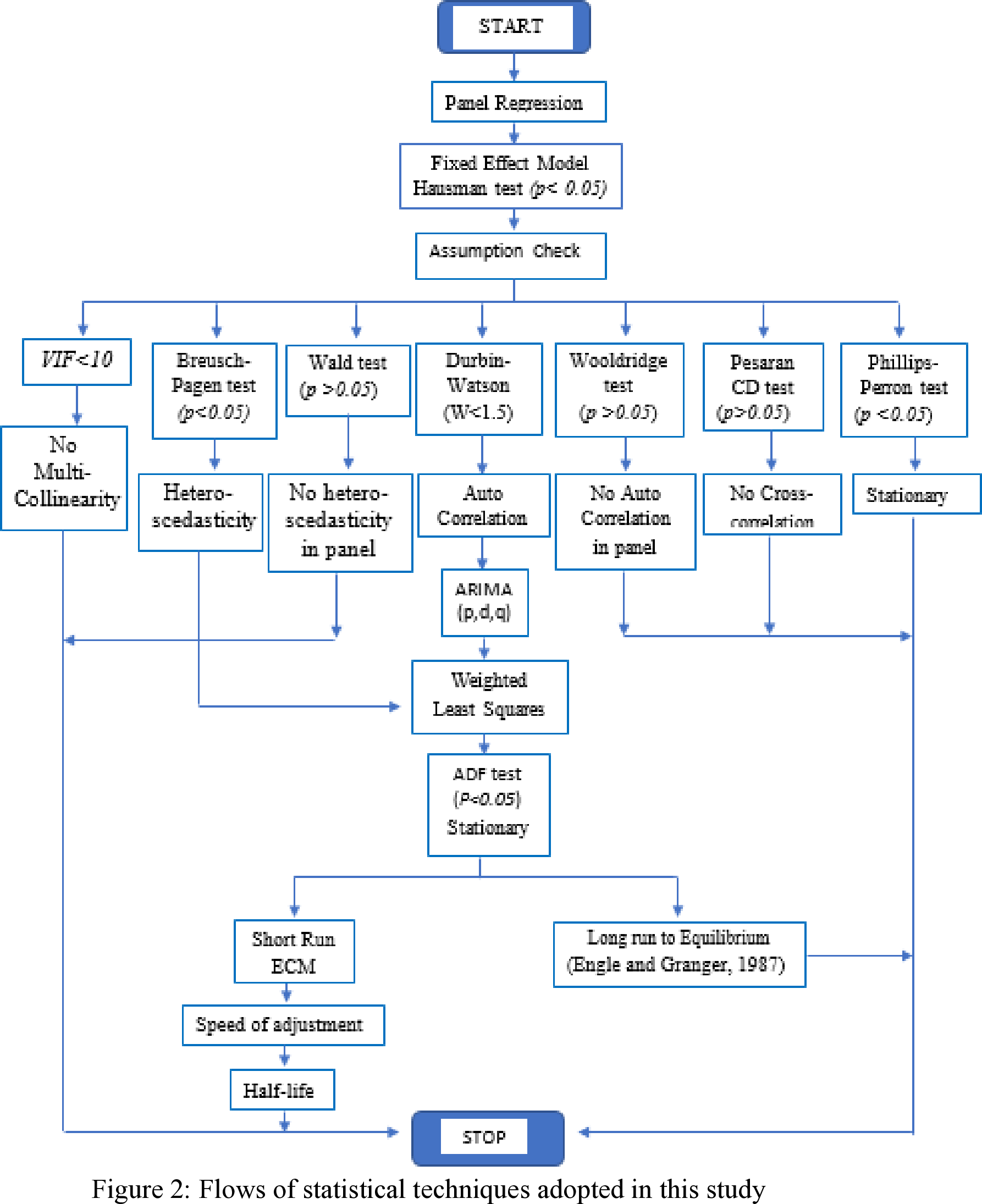

Supplement: S2 Fig — (TIF) [file pone.0316478.s002.tif]
