# Supplementary material for: Analyzing robust dividend payout policy with dynamic panel regression: Application of speed of adjustment to half-life
Source: PLoS One. 2025 Jan 15;20(1):e0316478. doi: 10.1371/journal.pone.0316478 (PMC11734913; doi:10.1371/journal.pone.0316478)
Supplement: Attachment — Submitted filename: Review 42377.docx [file pone.0316478.s004.docx]

**Review**

Dear authors,

This research article provides a well-rounded background on the complexities and importance of dividend payout policy in the context of corporate finance. The study is commendable for recognizing both financial and operational factors, such as asset turnover, firm age, and GDP growth, which are often overlooked in the dividend payout policy literature. Additionally, the introduction of advanced statistical techniques like ARIMA, Weighted Least Squares, and the use of instrumental variables for handling endogeneity reflects a sophisticated approach. By targeting emerging economies and using a variety of variables, the research fills notable gaps in our understanding of dividend policy in diverse economic contexts. However, a few critiques may be considered:

1. Although ARIMA, WLS, and IV techniques are robust for addressing endogeneity, relying on ARIMA and WLS models might not sufficiently capture all nuances in panel data, especially when dealing with cross-sectional variations. More advanced econometric techniques, like dynamic panel GMM, might offer further insights in handling panel data complexities.
2. The study mentions the dividend signaling theory and accounting information relevance theory; however, it lacks a deeper discussion of how these theories interact with the empirical model. A more elaborate theoretical framework could provide clearer motivation for selecting specific variables.
3. While the paper provides a solid academic perspective, it would benefit from a stronger focus on the implications for corporate managers and policymakers in emerging economies, beyond general recommendations.
4. The study indeed falls short in articulating its unique contributions to the existing body of research on dividend payout policy. Without a clear discussion on how it advances or diverges from prior studies, the study may struggle to demonstrate its significance within the field.

Overall, while the research is thorough, it could be further enriched by expanding the theoretical foundation, refining the methodology, and enhancing practical applications.
