# Supplementary material for: Analyzing robust dividend payout policy with dynamic panel regression: Application of speed of adjustment to half-life
Source: PLoS One. 2025 Jan 15;20(1):e0316478. doi: 10.1371/journal.pone.0316478 (PMC11734913; doi:10.1371/journal.pone.0316478)
Supplement: Attachment — Submitted filename: response to reviwers.docx [file pone.0316478.s005.docx]

**Authors’ Reply**

**Reviewer #1:**

1. Introduction: The introduction is well outlined. However, "Problem Statement" and "Objective" of the article need to be clearly defined.

Revised as recommended. (pages 2 and 3)

2. Literature Review: The review of empirical research is well explained with critical justification. Underlying theories were also explained. However, a clear literature gap is still shallow in the description. The author is advised to clearly identify and outline the literature gap.

Revised as recommended. (pages 6 and 7)

3. Conceptual Framework: The conceptual Framework is developed. In addition, the author is advised to include the hypotheses.

Thank you for the suggestion to include hypotheses in the manuscript. While we appreciate the importance of clearly stating hypotheses in many research contexts, this study is structured around a data-driven exploration of financial information and previously successful factors. This approach allows for a broader examination of these relationships, beyond a confirmatory framework, and is consistent with our research objectives. Therefore, we have opted to focus on the empirical findings and interpretations without predefined hypotheses to maintain the exploratory nature of our study.

4. Research Methodology: Excellent and rigorous statistical analysis techniques were followed.

Thank you.

5. Findings: Reasonably well written technical analysis on findings. No further discussion is required.

Thank you.

6. Discussion: Excellent discussions on theoretical and practical contributions, limitations and scope for further research. No edit is required.

Thank you.

**Review#2**

Dear authors,

This research article provides a well-rounded background on the complexities and importance of dividend payout policy in the context of corporate finance. The study is commendable for recognizing both financial and operational factors, such as asset turnover, firm age, and GDP growth, which are often overlooked in the dividend payout policy literature. Additionally, the introduction of advanced statistical techniques like ARIMA, Weighted Least Squares, and the use of instrumental variables for handling endogeneity reflects a sophisticated approach. By targeting emerging economies and using a variety of variables, the research fills notable gaps in our understanding of dividend policy in diverse economic contexts. However, a few critiques may be considered:

1. Although ARIMA, WLS, and IV techniques are robust for addressing endogeneity, relying on ARIMA and WLS models might not sufficiently capture all nuances in panel data, especially when dealing with cross-sectional variations. More advanced econometric techniques, like dynamic panel GMM, might offer further insights in handling panel data complexities.

Thank you for your insightful comments regarding our methodological approach. We recognize that dynamic panel GMM offers robust tools for addressing certain aspects of panel data, especially with cross-sectional variations and endogeneity. However, in this study, we selected ARIMA, WLS, and IV techniques to address endogeneity and autocorrelation, with an emphasis on model simplicity and interpretability in line with our specific data structure and research objectives.

Panel data, which combines cross-sectional and time series dimensions, is often associated with endogeneity issues. Endogeneity can lead to challenges such as autocorrelation and heteroscedasticity. While we acknowledge that GMM is a robust method to address endogeneity, it is important to note that these problems can also be effectively managed through alternative techniques. For instance, autocorrelation can be addressed using methods such as Generalized Least Squares, Exponential Smoothing, Spectral Analysis, Fourier Analysis, or ARIMA (Wiboonpongse et al., 2015; Barros et al., 2015). Similarly, heteroscedasticity can be mitigated with approaches like Weighted Least Squares (WLS), Heteroscedasticity-Consistent Standard Errors (HGL), and Driscoll-Kraay standard errors (Gill, 2007; Hoechle, 2007; Wooldridge, 2007). These techniques aim to ensure that estimators possess desirable properties, particularly being BLUE (Best Linear Unbiased Estimator).

While GMM is also powerful, we believe that our approach is well-suited for capturing the primary dynamics relevant to our analysis. Nonetheless, we mention this suggestion in future work to further enhance robustness. (page 25)

Refrrences:

Barros, L. A., Bergmann, D. R., Castro, F. H., & Silveira, A. D. M. D. (2020). Endogeneity in panel data regressions: methodological guidance for corporate finance researchers. *Revista brasileira de gestão de negócios*, *22*, 437-461.

Hoechle, D. (2007). Robust Standard Errors for Panel Regressions with Cross-Sectional Dependence. The Stata Journal, 7, 281-312

Gill, J., & Leemann, L. (2001). Weighted Least Squares. In Generalized Linear Models: A Unified Approach (pp. 42-44). SAGE, Thousand Oaks, California.

Wiboonpongse, A., Liu, J., Sriboonchitta, S., & Denoeux, T. (2015). Modeling dependence between error components of the stochastic frontier model using copula: Application to intercrop coffee production in Northern Thailand. International Journal of Approximate Reasoning, 65, 34-44.

Wooldridge, J. M. (2010). Econometric analysis of cross section and panel data. MIT Press, Cambridge.

2. The study mentions the dividend signaling theory and accounting information relevance theory; however, it lacks a deeper discussion of how these theories interact with the empirical model. A more elaborate theoretical framework could provide clearer motivation for selecting specific variables.

Revised as recommended (pages 4 and 5).

3. While the paper provides a solid academic perspective, it would benefit from a stronger focus on the implications for corporate managers and policymakers in emerging economies, beyond general recommendations.

Revised as recommended (page 22).

4. The study indeed falls short in articulating its unique contributions to the existing body of research on dividend payout policy. Without a clear discussion on how it advances or diverges from prior studies, the study may struggle to demonstrate its significance within the field.

Revised as recommended (page 23).
